# Optimal combined anteversion range for obtaining a wider range of motion without prosthetic impingement after total hip arthroplasty: a three-dimensional analysis study

**DOI:** 10.1186/s13018-022-03112-6

**Published:** 2022-04-10

**Authors:** Ryo Hidaka, Kenta Matsuda, Masaki Nakamura, Shigeru Nakamura, Hirotaka Kawano

**Affiliations:** 1 Department of Orthopaedic Surgery, Teikyo University School of Medicine, 2-11-1, Kaga, Itabashi-ku, Tokyo, 173-8606 Japan; 2 Department of Orthopaedic Surgery, Toranomon Hospital, 2-2-2, Toranomon, Minato-ku, Tokyo, 105-8470 Japan; 3 Department of Orthopaedic Surgery, Teikyo University Mizonokuchi Hospital, 5-1-1, Futako, Takatsu-ku, Kawasaki, Kanagawa 213-8507 Japan

**Keywords:** Activities of daily living, Arthroplasty, Hip prosthesis, Joint dislocation, Polyethylene, X-ray computed tomography

## Abstract

**Background:**

Obtaining a larger theoretical range of motion (ROM) is crucial to avoid prosthetic impingement after total hip arthroplasty (THA); however, no reports have examined the permissible range values of combined anteversion (CA) satisfying targeted ROM without prosthetic impingement. This retrospective study aimed to evaluate the possible postoperative CA extent that would allow meeting target ROM criteria according to Yoshimine’s theory using computed tomography (CT)-based three-dimensional motion analysis after THA.

**Methods:**

This study included 114 patients (133 hips) who underwent cementless primary THA using a CT-based navigation system and implants (oscillation angle ≥ 135°). Implant positions were determined using Yoshimine's CA formula. Postoperative evaluation was conducted using a three-dimensional templating software for CT data. The postoperative Yoshimine’s and Widmer’s CA was calculated, and the difference between the target and postoperative values was defined as the error of Yoshimine’s CA and Widmer’s CA. Prosthetic ROM was assessed by Yoshimine’s stringent criteria for activities of daily living. Based on fulfilling these criteria, all patients were divided into the ROM (+) and ROM (−) groups. Evaluation items were compared between the two groups.

**Results:**

There were 111 and 22 hips in the ROM (+) and ROM (−) groups, respectively. A significant difference was noted in the absolute error of Yoshimine’s and Widmer’s CA between the two groups. Using receiver operating characteristic analysis, threshold values of 6.0 (higher values indicate greater disability; sensitivity 90.9%, specificity 72.1%) for the absolute Yoshimine’s CA difference (area under the curve [AUC] 0.87, *P* < 0.01) and 6.9 (higher values indicate greater disability; sensitivity 68.2%, specificity 88.3%) for the absolute Widmer’s CA difference (AUC 0.83, *P* < 0.01) were predictors in the ROM (−) group.

**Conclusions:**

The target range of Yoshimine’s CA (90.8° ± 6.0°) and Widmer’s CA values (37.3° ± 6.9°) was crucial in implant orientation for obtaining theoretical ROM without prosthetic impingement after THA.

## Background

Impingement between the cup and femoral neck causes dislocation, pain, polyethylene liner wear, and component loosening after total hip arthroplasty (THA). The optimum orientation of implants is important to obtain a wider range of motion (ROM) and to avoid impingement between prosthetic components. Combined anteversion (CA) is one of the indexes widely used in cup–stem orientations. CA, the sum of the cup and stem anteversion, has been recommended to be between 25° and 35° in men and up to 45° in women [1]. The dislocation risk was reportedly 6.9 times greater if CA fell outside the range of 40°–60° after THA [2]. Another report recommended that CA should be within 40°–60° to reduce dislocations after THA using the CA technique [3]. Therefore, the generally accepted CA has been reported to range from 40° to 60°. There have been few reports on the target CA value for establishing the criteria for ROM without prosthetic impingement based on activities of daily living analyzed by three-dimensional simulation and mathematical formulas [4, 5]. Widmer et al. [4] reported the following formula for the target CA value using a three-dimensional computer model/cup radiographic anteversion + 0.7 × stem anteversion = 37.3°.

This was obtained when the cup radiographic inclination was between 40° and 45° according to the following six ROM conditions: flexion ≥ 130°, internal rotation ≥ 80°, extension ≥ 40°, external rotation ≥ 40°, abduction ≥ 50°, and adduction ≥ 50°. Yoshimine et al. [5] reported a similar analytical approach to these mathematical formulas and indicated four ROM conditions of flexion > 120°, internal rotation at 90° flexion > 45°, extension > 30°, and external rotation > 40°. In addition, their study showed that the CA value to fulfill their criteria could be determined using the formula: cup radiographic inclination + cup anatomical anteversion + 0.8 × stem anteversion = 90.8°. These were simulation studies. However, in clinical practice, postoperative implant positions often could not be accurately reproduced according to preoperative planning. The use of navigation systems has improved the implant placement accuracy, making it possible to place implants closer to the target CA with less error [6, 7]. However, to the best of our knowledge, no study to date has reported the degree of error that is allowed from the target CA value to apply these CA theories to THA to meet the ROM criteria.

Therefore, the purpose of our study was to evaluate the extent to which postoperative CA could be allowed to meet the target ROM criteria according to Yoshimine’s CA theory using computed tomography (CT)-based three-dimensional motion analysis after THA.

## Methods

**Fig. 1:**
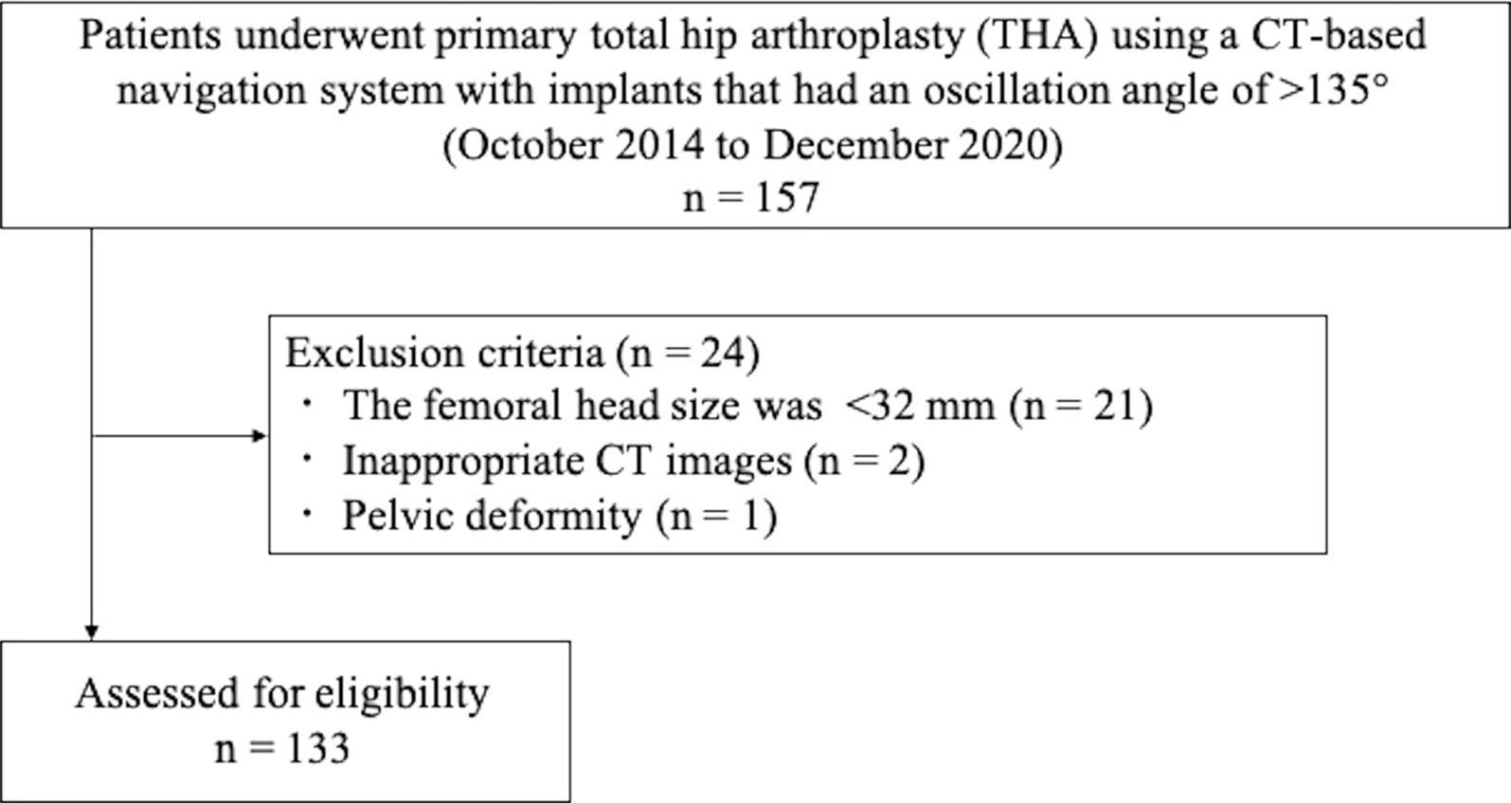
Patient selection flowchart

The study was approved by the institutional review board (approval number: 21–086). This retrospective case-series study enrolled patients who underwent cementless primary THA using a CT-based navigation system (Stryker CT-Hip System V1.1, Stryker-Leibinger GmbH & Co. KG, Freiberg, Germany) at Teikyo University Hospital between October 2014 and December 2020. We perform approximately 100 such operations per year at our institution. Inclusion criterion was the use of implants with an oscillation angle of > 135° according to Yoshimine’s CA theory. The two types of components were included: (1) G7 cup and Taperloc complete stem (Zimmer Biomet Holdings, Inc., Warsaw, IN, USA) and (2) SQRUM cup and J-Taper stem (Kyocera, Inc., Osaka, Japan). Exclusion criteria were a head size < 32 mm not meeting an oscillation angle of > 135° according to Yoshimine’s CA theory, patients with inappropriate CT images, and patients with a pelvic deformity that could not be set at the pelvic coordinate system. This study retrospective reviewed collected data from 133 hips of 114 patients (Fig. 1).

### Preoperative planning

A preoperative CT scan extending from the iliac wing to the knee joint was obtained using a helical CT scanner (Light-Speed VCT; GE Medical Systems, Milwaukee, WI, USA). All imaging data were transferred to a CT-based three-dimensional templating system for preoperative planning (ZedHip™ Lexi Co., Ltd., Tokyo, Japan). The pelvic coordinate system was the anterior pelvic plane, which was defined by the anterior superior iliac spine and the pubic tubercle. The femoral coordinate system was defined by the posterior condylar plane of the femur, which was formed by the proximal posterior surface, lateral condyle, and medial condyle. The size and anteversion of the stem were adjusted to match the shape of the proximal femoral medullary canal. Stem anteversion was defined as the angle formed between the proximal femoral stem axis and the line tangential to the bilateral posterior femoral condylar margin on the axial plane. A modular stem was selected for the hips with an anticipated stem anteversion of < 10° or > 40° in preoperative planning. Non-modular stems were used in all the enrolled hips in anticipation of stem anteversion in the range of 10°–40°. The target cup radiographic inclination was determined to be 43° in all the hips. Cup anteversion was calculated for each hip using the following formula: cup radiographic inclination + cup anatomical anteversion + stem anteversion × 0.8 = 90.8°. The planning data were entered into the CT-based navigation system.

### Surgical procedure

All surgeries were performed using a posterolateral approach with the patient in the lateral decubitus position. A pelvic tracker was fixed percutaneously on the ipsilateral ilium using two 4-mm pins and an external fixation device (Hoffman II, Stryker-Leibinger GmbH & Co. KG, Freiberg, Germany). In addition, surface matching of the pelvis was completed by digitizing more than 30 points around the acetabulum. The acetabular cup was implanted using a navigation system, and the stem was manually placed according to the anatomy of the proximal femur. All surgeries were performed by either of the two senior authors.

### Postoperative evaluation

CT was performed 1–2 weeks postoperatively using a minimal radiation dose protocol [8]. Postoperative CT data were transferred to ZedHip for three-dimensional analysis. All preoperative planning and postoperative CT reference points were matched manually, and implant orientation was measured. This software was capable of simulating and detecting implant impingement, which allowed the maximum prosthetic ROM to be defined as the number of degrees of movement before the occurrence of implant impingement.

### Measurements

**Fig. 2:**
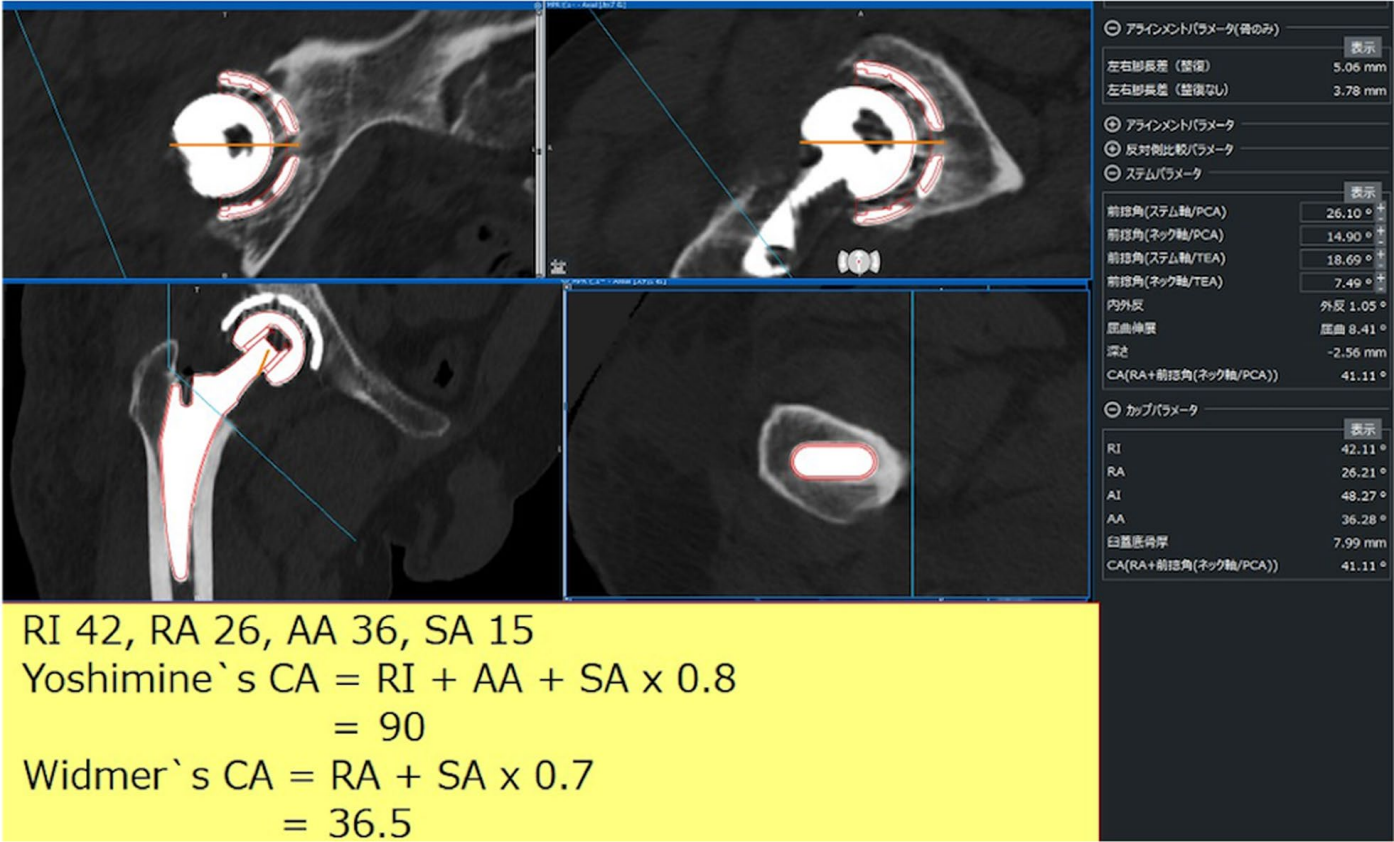
Implant orientations are measured by superimposing the CAD models of the implants on postoperative CT images using Zed Hip. Yoshimine’s CA and Widmer’s CA were calculated from the implant orientation measurements. CAD, computer-aided design; *CA* combined anteversion

All measurements were assessed by one author (RH). Postoperative Yoshimine’s CA and Widmer’s CA were calculated from the implant orientation measurements (Fig. 2). The difference between the target CA value (90.8°) and the postoperative Yoshimine’s CA was defined as the error of Yoshimine’s CA. The difference between the target CA value (37.3°) and the postoperative Widmer’s CA was defined as the error of Widmer’s CA. The difference between preoperative and postoperative values in each CA component, cup radiographic inclination, cup anatomic anteversion, and stem anteversion was also calculated. In ROM-simulation analysis, the neutral hip position of our system was defined as the position in which all corresponding axes in the pelvic and femoral coordinate systems were parallel. Moreover, the prosthetic ROM in those directions was measured using a previously reported method [9]. Several reports have shown that the ROM was affected by the femoral offset and head diameter [10–13]; therefore, in all cases, the femoral offset was standardized to a stem with a standard offset type and the minimum neck length for each stem. The femoral head diameter was standardized to 32 mm, and the prosthetic ROM was measured.

The offset of the Taperloc complete stem (neck shaft angle 133°, cone 12/14 taper) and J-Taper stem (neck shaft angle 130°, cone 9/10 taper) increases with stem size and ranges from 31.2 to 37.7 mm and 32 to 40 mm, respectively, for the standard offset geometry.

We investigated whether all directions were fulfilled based on Yoshimine’s stringent ROM conditions for activities of daily living (flexion > 120°, internal rotation > 45° at flexion of 90°, external rotation > 40° at flexion 0°, and extension > 30°). Patients who fulfilled all criteria were classified into the ROM (+) group and patients who did not fulfill the criteria were classified into the ROM (-) group.

All hips were followed for at least 1 year, during which, any complications were recorded.

### Statistical analysis

Demographic variables are presented as mean, standard deviation, and range for continuous data. Fisher’s exact test was used to compare categorical parameters, and the Wilcoxon rank-sum test was used to compare continuous parameters between the groups. Univariate analysis was conducted on each possible factor to screen for significant factors related to the ROM (−) group. Optimal threshold scores for the absolute error of Yoshimine’s CA and Widmer’s CA that predicted the ROM (−) group were calculated using a nonparametric receiver operating characteristic (ROC) analysis. Youden's index was used to calculate the optimal threshold scores to obtain the best balance of sensitivity and specificity [14]. The significant risk factors identified by univariate analysis were examined by logistic regression analysis to determine their contribution to the ROM (−) group. We defined statistical significance at 5% (*P* < 0.05) level. Statistical analyses were performed using SPSS 24.0 software (IBM Corp., Armonk, NY, USA).

## Results

**Fig. 3:**
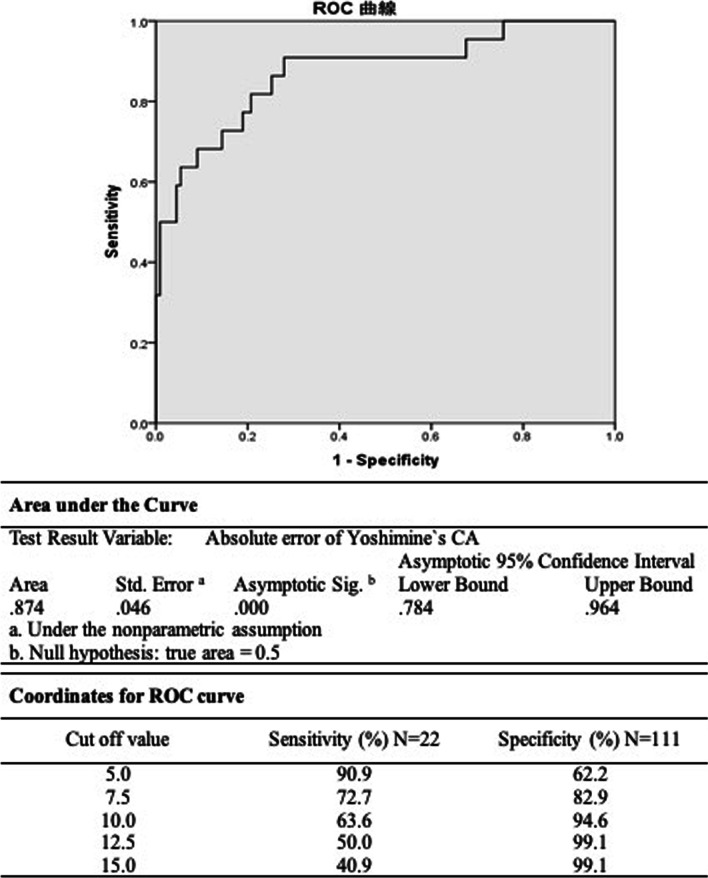
ROC curve on the predictive value of absolute error of Yoshimine’s CA in the ROM (−) group ROC, receiver operating characteristic; *CA* combined anteversion

**Fig. 4:**
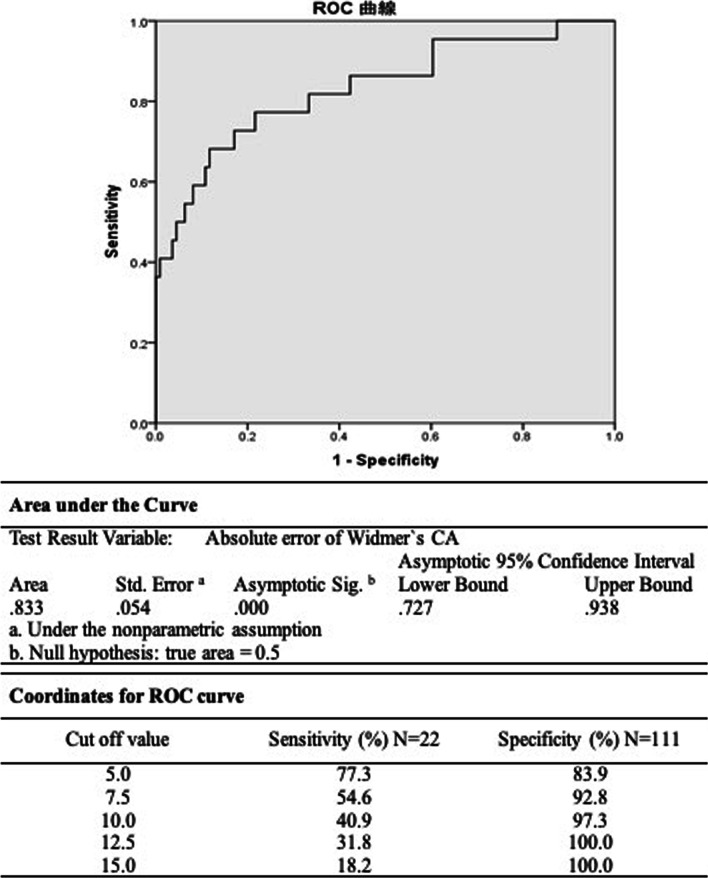
ROC curve on the predictive value of absolute error of Widmer’s CA in the ROM (−) group ROC, receiver operating characteristic; *CA* combined anteversion

The patients’ demographics and implant size are summarized in Table 1. There were 111 hips in the ROM (+) group and 22 hips in the ROM (−) group. The following details were noted for the ROM (−) group: 1 hip (120°) in flexion, 6 hips (range 33°–45°) in internal rotation at flexion of 90°, 9 hips (range 30°–40°) in external rotation, 2 hips (range 27°–29°) in extension, 1 hip (115° and 45°) in flexion and internal rotation at flexion of 90°, and 3 hips (range 28°–36° and 26°–29°) in external rotation and extension. Univariate analysis indicated that there was a significant difference in age, sex, and absolute error of Yoshimine’s CA and Widmer’s CA between the two groups (Table 2); however, there was no significant difference in Yoshimine’s CA and Widmer’s CA. In ROC analysis, a threshold value of 6.0 (higher values indicate greater disability; sensitivity 90.9%, specificity 72.1%) for the absolute error of Yoshimine’s CA was predictive of the ROM (−) group (AUC 0.87, *P* < 0.01) (Fig. 3), and a threshold value of 6.9 (higher values indicate greater disability; sensitivity 68.2%, specificity 88.3%) for the absolute error of Widmer’s CA was predictive of the ROM (−) group (AUC 0.83, *P* < 0.01) (Fig. 4). Multivariate analysis demonstrated that the absolute error of Yoshimine’s CA was significantly associated with the ROM (−) group, with an adjusted OR of 1.47 (95% confidence interval 1.25–1.71, *P* < 0.01) (Table 3). Other factors were not associated with the ROM (−) group. Three cases of posterior hip dislocation and two cases of early infection were noted during the follow-up. One case of posterior hip dislocation required revision surgery.

**Table 1:** Patient demographics

Demographics	Values
Number of patients (hips/patients)	133/114
Sex (male/female)	32/82 patients
Age (years)^a^	64 ± 11 (39–84)
BMI (kg/m^2^)^a^	24 ± 4 (16–40)
Diagnosis (hips)	
Osteoarthritis	102
Osteonecrosis of the femoral head	24
Femoral neck fractures	5
Rheumatoid arthritis	1
Cup size	50 (46–62)
Stem size	
Taperloc stem	9 (4–17)
J-Taper stem	6 (1–11)

*BMI* body mass index

^a^Values are given as mean ± standard deviation (SD) (range)

**Table 2:** Univariate analysis of the factors for each range-of-motion [ROM ( +) and ROM (−)] group

Factors	Total N = 133	ROM ( +) N = 111	ROM (−) N = 22	*P* values
Age (years) ^a^		63.4 ± 11.0	59.2 ± 9.2	0.03
Sex (hips)				
Male	41	29	12	0.01
Female	92	82	10	
BMI (kg/m^2^) ^a^		24.4 ± 4.2	23.8 ± 3.7	0.71
Diagnosis (hips)				
OA	101	88	15	0.12
ONFH	24	18	6	
FNF	5	5	0	
RA	1	0	1	
Implants (hips)				
G7-Taperloc	80	68	12	0.63
SQRUM-J-taper	53	43	10	
Yoshimine`s CA (degrees) ^a^	91.1 ± 7.4	90.5 ± 5.6	93.6 ± 13.1	0.06
Widmer`s CA (degrees) ^a^	38.0 ± 5.6	37.6 ± 4.3	40.2 ± 9.8	0.06
Absolute error of Yoshimine`s CA (degrees) ^a^	5.7 ± 4.6	4.5 ± 3.2	11.9 ± 5.5	< 0.01
Absolute error of Widmer`s CA (degrees) ^a^	4.4 ± 3.6	3.5 ± 2.5	8.8 ± 4.8	< 0.01
Each factors of CA				
Stem anteversion (degrees) ^a^	5.4 ± 4.4	5.1 ± 4.0	6.9 ± 5.5	0.18
Cup radiographic inclination (degrees) ^a^	3.1 ± 2.3	3.0 ± 2.3	3.7 ± 2.4	0.17
Cup anatomical anteversion (degrees) ^a^	4.3 ± 3.6	3.9 ± 3.1	6.0 ± 5.3	0.08

*BMI* body mass index, *OA* osteoarthritis, *ONFH* osteonecrosis of the femoral head, *FNF* femoral neck fractures, *RA* rheumatoid arthritis, *CA* combined anteversion

^a^Values are given as mean ± standard deviation (SD) (range)

**Table 3:** Multivariate analysis of the factors for the range-of-motion (−) group

Factors	Odds ratio	95% confidence interval	*P* value
Age	0.97	0.91–1.03	0.26
Sex	0.34	0.09–1.31	0.12
BMI	0.90	0.76–1.07	0.23
Absolute error of Yoshimine’s CA	1.47	1.25–1.71	< 0.01

*BMI* body mass index, *CA* combined anteversion

## Discussion

CA is widely used as an index of implant orientation. To the best of our knowledge, no study to date has examined the permissible range of CA values to satisfy the ROM criteria for activities of daily living without prosthetic impingement with CA after THA. This study demonstrated that the range of postoperative Yoshimine’s CA and Widmer’s CA satisfied the ROM conditions for activities of daily living after THA.

Few studies have evaluated ROM on simulation after THA clinically, as in this study. One study has shown impingement-free ROM after THA with the stem-first technique using imageless navigation [15]. Their ROM criteria to be satisfied were defined as: flexion > 110°, internal rotation at a flexion 90° > 30°, extension > 30°, abduction > 45°, abduction > 50°, and adduction > 30°. Out of the 57 cases using imageless navigation, 48 (84%) met all the ROM criteria. The ROM criteria were different from the strict criteria in our study, except for external rotation. However, 83.5% (111/133 hips) of the cases in our study met our ROM criteria. This report has not evaluated implant orientation before or after surgery and the target CA and did not indicate the range of postoperative CA to meet the ROM criteria. We evaluated the impingement-free ROM by postoperative CT after THA with a cup-first technique using CT-based navigation targeted by Yoshimine’s CA, according to a previous study [16]. We reported that 45 (88%) of the 51 hips met all the Yoshimine’s ROM criteria. Moreover, postoperative Yoshimine’s CA within the target range of 90.8 ± 10 would significantly meet all ROM criteria. However, we have not examined whether postoperative Yoshimine’s CA within 10° from the target value would be suitable as a range to meet the Yoshimine’s ROM criteria.

Yoshimine’s CA and Widmer’s CA theories recommended a target CA value of 90.8 and 37.3. Given that navigation systems have potential to implant components in an optimal orientation, there have been many reports on the accuracy of implant orientation in THA using these navigation systems. Inaba et al. [7] reported absolute differences between preoperative and postoperative cup inclination as 3.2° ± 2.3°, cup anteversion 4.0° ± 3.5°, stem anteversion 3.9° ± 5.0°, and Widmer’s CA 5.3° ± 5.2° using a CT-based navigation system. Dorr et al. [17] showed that the average CA was 37.6° ± 7.0°, and 45 cases (96%) out of 47 were within the target CA of 25°–50° using an imageless navigation system. Although tools such as navigation systems were used for accurate implant orientation, errors may occur between preoperative and postoperative implant orientation. It has been difficult to place components to set target CA accurately during THA. Therefore, we consider that it is important to examine the range of postoperative CA obtaining the target ROM criteria to be indexes evaluated for implant orientation after surgery and to set an implant position intraoperatively. This study revealed that the absolute value of difference between the target and postoperative values of Yoshimine’s CA was a significant factor in whether or not the ROM criteria were met using univariate and multivariate analyses. This study showed that postoperative Yoshimine’s CA and Widmer’s CA should be within 90.8° ± 6.0° and 37.3° ± 6.9°, which is likely to meet the Yoshimine’s ROM criteria for activities of daily living. Our results indicated a useful target range of Yoshimine’s CA and Widmer’s CA in considering the error related to implant orientation in THA.

The absolute error of Yoshimine’s CA and Widmer’s CA was 5.7 ± 4.6 and 4.4 ± 3.6, respectively, in our study, which is comparable to Widmer’s CA 5.3° ± 5.2° of previous studies using CT-based navigation [7]. The use of CT-based navigation could predict whether the postoperative CA will place the implant within the target range of CA.

There were two cases in which the prosthetic ROM did not meet the Yoshimine’s ROM criteria, even though the Yoshimine’s CA was in the range of 90.8° ± 6.0°. The Yoshimine’s CA in two cases was 88.5° and 92.6°. These cases did not reach the ROM boundary only for one direction each of internal rotation at flexion 90° and external rotation. These cases did not reach the ROM benchmark due to a difference of 1°. Three-dimensional templating software has been shown to have excellent interobserver and intraobserver reliability for component alignment in THA [18]. Inoue et al. [19] reported that error measurement might be performed using this software when manually matching the reference points between the preoperative plan and postoperative evaluation on CT. Two studies have reported that the sagittal alignment of the stem had an influence on impingement-free ROM [20, 21]. Therefore, it was considered that a measurement error with the three-dimensional templating software occurred, which influenced the sagittal alignment of the stem.

This study has several limitations. First, we excluded the cases wherein the stem anteversion was > 40° or < 10°. For cases wherein, the stem anteversion was > 40°, cup radiographic anteversion was set to < 10°, according to the CA theory, and if errors occurred in these cases, cup radiographic anteversion may have led to retroversion. Widmer et al. [4] did not recommend cup radiographic anteversion of < 10° to be incompatible with the intended ROM. When stem anteversion was < 10°, cup radiographic anteversion was > 30° and the acetabulum could not sufficiently cover the posterior area of the cup. A cementless stem was implanted manually without the use of navigation systems in this study. Anteversion of the cementless stem was hard to control because of the anatomy of the proximal femur. A broad range of postoperative stem anteversion has been reported in the literature [22–24]. The postoperative error may cause the stem anteversion to become retroverted. Dorr et al. [17] reported similar results showing that 2 of 47 hips exhibited postoperative CA of < 25° due to the retroverted native femoral anteversion that was not in the safe zone. Therefore, stem anteversion should be avoided.

Second, two types of implant designs and oscillation angles were included. The formula of Yoshimine’s CA was calculated based on the assumption that the implant design had an oscillation angle of 135° [5]. The study showed that the safe zone for an oscillation angle of 120° was extremely small, and implant designs with a greater oscillation angle were recommended. Two implant designs had an oscillation angle of ≥ 135° in our study. Many varieties of implant designs have been used widely in THA. Our study showed that the use of two types of implants with an oscillation angle of ≥ 135° could fulfill the Yoshimine’s ROM criteria within the range of ± 6.0° according to Yoshimine’s CA. However, this study may not be accurate when using implants with an oscillation angle of ≤ 135°. Third, this simulation study investigated only ROM without prosthetic impingement. Clinically, ROM without bone and soft-tissue impingements was also a risk factor of dislocation. However, we did not evaluate them. The factors that the surgeon could control to avoid dislocation were to place the optimum implant positions and to increase prosthetic ROM.

## Conclusions

It is ideal to place implants according to CA closer to the target value for meeting the ROM criteria according to CA theory. However, the target and postoperative CA values can be different due to an error in implant placement during surgery. In this study, if postoperative Yoshimine’s CA and Widmer’s CA were within 6.0° and 6.9°, respectively, from the target CA value after THA, the potential ROM without implant impingement could meet Yoshimine’s ROM criteria for activities of daily living. This range of CA values could serve as an index for postoperative implant orientations. This may reduce problems caused by implant impingement, such as dislocation.

## Data Availability

The datasets used and analyzed during the current study are available from the corresponding author on reasonable request.

## Abbreviations

THA: Total hip arthroplasty
ROM: Range of motion
CA: Combined anteversion
CT: Computed tomography
ROC: Receiver operating characteristic
